# Association of Early Direct Bilirubin Levels and Biliary Atresia Among Neonates

**DOI:** 10.1001/jamanetworkopen.2019.13321

**Published:** 2019-10-16

**Authors:** Fatima Noorulla, Russell Dedon, M. Jeffrey Maisels

**Affiliations:** 1 Department of Pediatrics, Beaumont Children’s, Royal Oak, Michigan; 2 Oakland University William Beaumont School of Medicine, Rochester, Michigan

## Abstract

This case series evaluates the direct bilirubin levels in the first days after birth among neonates who were subsequently diagnosed with biliary atresia.

## Introduction

Biliary atresia (BA) is a fibrosclerosing cholangiopathy resulting in obstruction of the biliary tree. Diagnosis and early performance of the Kasai hepatic portoenterostomy improves outcomes.^1^ Current guidelines recommend measurement of direct bilirubin (DB) or conjugated bilirubin levels in neonates who are still jaundiced at age 2 to 3 weeks.^2,3^ However, recent studies have documented the elevation of DB or conjugated bilirubin levels within the first days after birth in neonates with BA,^4,5^ suggesting that BA is a developmental cholangiopathy associated with a genetic, prenatal, or developmental event. To test this hypothesis, this case series evaluated DB levels in the first days after birth among neonates who were subsequently diagnosed with BA.

## Methods

We performed a retrospective review of all patients diagnosed with BA from 2 hospitals in the Beaumont Health System in Royal Oak, Michigan, and Troy, Michigan, from January 1992 to July 2018. We included only patients whose DB levels were measured at a Beaumont facility within the first week of life, whose liver biopsy results were consistent with BA, and who underwent a Kasai hepatic portoenterostomy. This study was approved with a waiver of consent by the Beaumont Health System institutional review board because the research involved data that had been collected solely for nonresearch purposes. This study followed the reporting guideline for case series.

We used Excel 2011 spreadsheet software (Microsoft Corp) to organize our data and calculate percentiles. Data were analyzed from August to September 2018.

## Results

The median (range) total serum bilirubin (TSB) level in the population was 11.9 (4.2-17.6) mg/dL (to convert to micromoles per liter, multiply by 17.104), and the median (range) DB level was 1.7 (0.8-4.9) mg/dL. From DB measurements in 10 652 neonates, we established 1.0, 1.1, and 1.3 mg/dL as our 95th, 97.5th, and 99th percentiles, respectively (data not shown). We identified 8 neonates with BA (6 female; 2 male; gestational age, 34-41 weeks), all of whom had a DB level higher than 1.3 mg/dL (ie, 99th percentile) within the first week (Figure). In 1 neonate, the DB level was 0.8 mg/dL (85th percentile) at age 8 hours and 1.0 mg/dL at 30 hours. Repeated measurements of DB levels were taken 10 to 104 hours later in 7 neonates and 1 month later in 1 neonate. Levels increased in 7 of 8 neonates (86%). Only 2 of 8 neonates (25%) had an initial DB-to-TSB ratio greater than 0.2.

**Figure.**
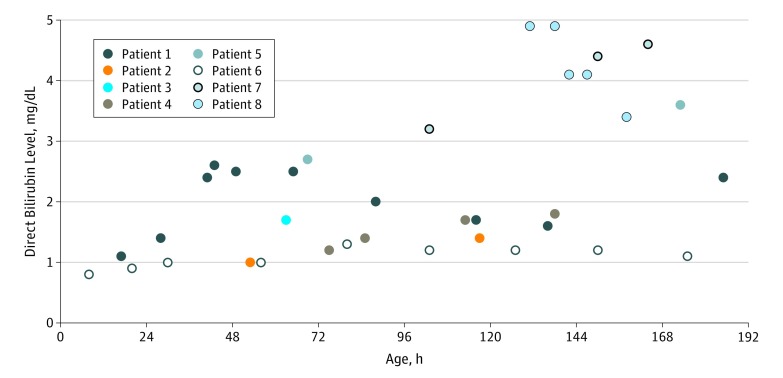
Direct Bilirubin Levels by Age in Hours in 8 Patients To convert direct bilirubin level to micromoles per liter, multiply by 17.104.

## Discussion

Our study had limitations, including the retrospective design and a small sample size. We could also have missed some cases of BA and excluded others because there was no measurement of DB in the first week. Nevertheless, our findings confirmed published data documenting that neonates with BA manifest direct hyperbilirubinemia within the first week of life (and, in some cases, as early as the first day) and that DB levels increase over time. Harpavat et al^5^ found that all 35 patients with BA in their study had an elevated conjugated bilirubin or DB level within 60 hours of birth. Although previously considered relevant, a DB-to-TSB ratio of more than 20% is no longer recommended as a diagnostic test that suggests the presence of cholestatic jaundice.^2^ The North American and European Societies for Pediatric Gastroenterology Guideline recommended that “an elevated serum direct bilirubin level (direct bilirubin levels >1 mg/dL or 17 μmol per liter) warrants timely consideration for evaluation and referral to a pediatric gastroenterologist or hepatologist.”^2^ Most of our patients had initial DB-to-TSB ratios less than 0.2, which questions the clinical relevance of a ratio of 0.2 or greater as a screening modality for neonatal cholestasis.

## Conclusions

This study confirmed published data documenting that neonates with BA manifest cholestasis within the first week and as early as the first day of life, that the cholangiopathy in BA begins in utero or in the early perinatal period, and that DB levels in neonates with BA increase over time. As others have also noted,^5^ we found that in neonates with BA, DB levels are initially less than 20% of TSB and, if the DB level in a neonate is higher than 1.0 mg/dL, a DB-to-TSB ratio less than 20% does not rule out the possibility of cholestasis. These neonates should undergo 1 or more additional tests of DB levels. If the DB level does not decrease below 1.0 mg/dL, the neonate should be evaluated for any of the possible causes of neonatal cholestasis, including BA. Large, prospective studies are required to confirm the clinical relevance and cost-effectiveness of DB screening in neonates.
